# A Prospective Study of Bipolar Transurethral Resection of Prostate Comparing the Efficiency and Safety of the Method in Large and Small Adenomas

**DOI:** 10.1155/2015/251879

**Published:** 2015-12-07

**Authors:** Nikolaos Mertziotis, Diomidis Kozyrakis, Christos Kyratsas, Andreas Konandreas

**Affiliations:** ^1^Iaso General Hospital, 264 Messogeion Avenue, 15562 Athens, Greece; ^2^General Hospital of Volos, 134 Polimeri Street, 38222 Volos, Greece

## Abstract

Bipolar technology offers a new perspective in the treatment of BPH.* Purpose.* To present our experience with the TURis system (Olympus, Tokyo, Japan).* Materials and Methods.* From February 2011 till December 2013 in a prospective study, 93 patients were treated for BPH. They were evaluated with IPSS, QoL, uroflow (*Q*_max_), and residual urine (RU), preoperatively as well as 6 and 9 months postoperatively. Based on the prostate volume, the patients were divided into two groups: group A (*n* = 48) with prostates ≥ 75 cc and group B (*n* = 45) with smaller prostate glands. All patients underwent bipolar TURP or/and plasma vaporization.* Results.* The postoperative improvement for IPSS, QoL, *Q*_max_, and RU was statistically significant. The operation time was longer in group A in comparison with group B (*P* < 0.001). The former group also had higher infection and stricture formation rates; however, there was no statistical difference between the two groups.* Conclusions.* Treatment with the TURis constitutes an effective technique and can be offered to large prostates with results equivalent to those in small ones. Regarding safety, large adenomas treated with TURis are not at a higher risk for urethral stricture but their odds to develop urogenital infections are relatively higher compared to the smaller adenomas.

## 1. Introduction

Benign prostate hyperplasia (BPH) is a high prevalent disease among the middle aged/elderly male population. Even though it is poorly defined, it is encountered at a rate of approximately 50% in ages between 51 and 60 years [1]. Others report a prevalence of 26% in males during their fifth decade of life and up to 46% during their eighth decade [2]. Medical treatment, with a_1_ blockers and 5-a reductase inhibitors, offers good results to patients with mild to moderate symptoms, while, for those with more severe lower urinary tract symptoms, an interventional treatment is recommended. For many years, transurethral resection of the prostatic adenoma with monopolar electrocautery (M-TURP) has been the gold standard of surgical treatment due to its effectiveness and its durable results over time but its safety profile is not ideal [3–5]. Postoperative hemorrhage, blood clot retention, and urethral strictures are a few of the potential complications. The hyponatremia and TUR syndrome are associated with the irrigation of a nonconductive solution (e.g., glycine 1.5%, mannitol 5%) to distend the bladder during the monopolar prostatectomy [6–8]. Prolonged resection time makes patients vulnerable to electrolyte disorders [9] and, for safety reasons, prostates greater than 80–100 mL are excluded from adequate treatment with M-TURP in one single session [10, 11].

Several devices and techniques have been developed to overcome these limitations of M-TURP and the bipolar resection of the prostate (B-TURP) is one of them. This method uses normal saline solution 0.9%, as irrigation fluid, which has the advantage to eliminate the risk of TUR syndrome [12, 13]. This is because the absorption of the irrigation fluid by the vascular system of the prostate is clinically insignificant. The bipolar device is also considered to have an optimal haemostatic effect minimizing the postoperative hemorrhage [14, 15].

We herein present the clinical results of a prospective study, composed of BPH patients treated with the bipolar resectoscope in saline, and we are comparing the surgical results and the complications encountered in large prostates with those in smaller adenomas.

## 2. Material and Methods

From February 2011 till December 2013, 93 consecutive patients were treated by the same surgeon for BPH with the bipolar 26 F resectoscope OES Pro by Olympus, Tokyo, Japan, in saline. Electric current was delivered by the electrosurgical generator UES-40 SurgMaster. Resection of the prostate was performed using the loop resectoscope combined, in some cases, with vaporization of the adenoma using the plasma button device (TURis).

Before treatment, patient history was taken and clinical examination was performed on each patient, followed by IPSS and quality of life (QoL) questionnaire, transabdominal or transrectal ultrasonography of the urinary tract, and uroflowmetry test with residual urine (RU) echographic assessment. In a prospective follow-up, all these tests were routinely repeated in 6–9 month interval after the operation.

The criteria for surgical treatment were formed based on one or more of the following: high prostate symptom score (IPSS ≥ 20), poor BPH related quality of life (score 5 or 6), failure to respond to conservative treatment or recurrence of symptoms after conservative treatment, *Q*_max⁡_ ≤ 10 mL/sec, high postvoiding residual urine volume (≥ 200 mL), and urinary retention or patient's preference. Discontinuation of any antiplatelet or anticoagulative treatment was mandatory prior to surgery.

Aiming to perform a comparative analysis, the presurgical prostate volume established the criterion based on which patients were classified into two groups; group A was that of large prostates (≥75 mL) and group B was the one with prostates less than 75 mL and represented the control group of our study.

The two groups were preoperatively examined for statistical significant differences regarding the age, the prostate volume, the IPSS, the QoL, the maximum flow (*Q*_max⁡_), and the RU. The surgical outcome was expressed as the postsurgical improvement over the baseline (preoperative) values for each one of the IPSS, QoL, *Q*_max⁡_, and RU and a comparison of the results between the two groups was provided. Operation time, hospitalization, postsurgical catheterization, and complication rates were recorded for each group separately and the results were statistically analyzed.

The statistical analysis was performed using the Stata MP 10.1 (StataCorp LP, Texas, USA) software for windows. Normality was examined using the Shapiro-Wilk test. Comparison of the two groups was performed using Wilcoxon rank-sum test and* t*-test for values in abnormal and normal distribution, respectively. Statistical significance was defined as *P* < 0.05.

## 3. Results

The patients' characteristics are presented in Table 1. The mean age at presentation was 71.3 years (range: 46–92). The mean prostate volume was 60.98 mL (range: 43–185). The mean IPSS, QoL, *Q*_max⁡_, and RU were 18.2, 3.37, 8.44 mL/sec, and 167.71 mL. For both groups, the mean operation time was 63.26 min (range: 36–151), the mean duration of catheterization was 28.06 hours (range: 16–98), and the mean hospital stay was 32.01 hours (range: 22–75). The percentage of improvement for IPSS, QoL, *Q*_max⁡_, and RU was 47.41%, −56.67%, 101.07%, and −65.97%, respectively, and statistically significant improvement was noted (Table 2). Two patients were unable to void after surgery. One of them was reoperated on and the other was treated with intermittent catheterization for 6 months. The latter subsequently had a successful voiding without catheterization. Two nonfatal major cardiovascular complications were diagnosed: one myocardial infarction and one pulmonary embolism. Urethral strictures were identified in 8 patients and urogenital infections in 10. Nine patients complained of persistent symptomatology of the lower urinary tract, mainly storage symptoms, for more than 3 months and were treated with anticholinergic regimens. An overview of surgical complications is presented in Table 3.

At the imaging of the lower urinary tract, 48 out of the 93 patients (52%, group A) had prostates greater than 75 mL (mean: 92.13 mL, range: 75–185) while the rest 45 patients (48%, group B) had smaller prostates (mean 51.29 mL, range: 43–66). The preoperative characteristics of both groups are presented in Table 4. When statistically examined, the two groups were comparable in all the preoperative characteristics except for the prostate size and the peak flow in uroflowmetry. The percentage of postsurgical improvement for group A was −52.46%, −47.57%, 157.68%, and −65.03% for IPSS, QoL, *Q*_max⁡_, and RU, respectively, and −45.41%, −60.60%, 116.08%, and −65.82% for group B, respectively. The statistical analysis did not reveal any significant difference in the surgical outcome between the two groups. The operation time was longer in large prostates, but the bladder catheterization time and hospital stay were similar (Table 5). The complication rates are presented in Table 6 and although the urogenital infection rate of group A was much higher than that of group B, no statistical significance was revealed.

## 4. Discussion

Historically, Gyrus (ACMI Southborough, MA, USA) was the first manufacturer that incorporated bipolar technology into the resectoscope device, known as the PlasmaKinetic System (PKS). The prostatectomy was performed using normal saline 0.9% as the irrigant fluid, instead of a nonconductive solution, offering the advantage of minimal absorption by the open vessels and eliminating the risk of electrolytic disorders, particularly the serum sodium level drop [12, 16]. Later on, another bipolar resectoscope, manufactured by Olympus (SurgMaster device, TURis), was released into the market having similar advantages to those of PKS [17]. The use of two interchangeable electrodes, the resection loop and the mushroom shaped plasma button, allows a fast, complete, and precise resection of the adenomas [18].

Nowadays, bipolar technology is a safe and effective method to perform the transurethral prostatectomy. An early meta-analysis published in 2009 showed that the bipolar method had the same efficacy as the monopolar one, but the safety of the former technique was more favorable. In particular, the clot retention rate and the TUR syndrome risk were lower in the bipolar arm. Moreover, the irrigation and catheterization time were significantly shorter [19]. Another meta-analysis published 4 years later, despite the methodological limitations of the RCT incorporated in the study and the short follow-up period, came to similar conclusions, emphasizing once more the better safety profile (non-TUR syndrome, less clot retention, and blood transfusion) encountered in the bipolar arm [13]. Aiming to overcome any methodological flaws, a well-designed multicenter double-blind randomized trial that fulfilled the COCHRANE criteria for high quality trials was performed, comparing the bipolar AutoZone II 400 ESU with the M-TURP. Although the dilutional hyponatremia was diagnosed more frequently in the monopolar group, the TUR syndrome risk was similar in both arms (monopolar: 0.7% versus bipolar: 0%). The authors concluded that the improved safety profile of the B-TURP was only theoretical, bearing minimal clinical significance when the operation was performed by experienced surgeons [20].

Nevertheless, the number of publications that focused on the surgical outcome in large volume prostates is limited. In a case series of 4 patients with excessive prostate volumes (>160 mL), prostatectomy was performed with the Gyrus PK system. Despite the prolonged operation time, the percentage of complications was favorable regarding the hemoglobin level and serum sodium level drop. The hospitalization time was short (mean: 12 hours) and the catheter was removed after an average of 76 hours [21].

In a prospective randomized study with adenomas greater than 60 gr., the PK system was compared with the conventional M-TURP. The short term surgical outcome (IPSS, *Q*_max⁡_, and RU) was similar between the two groups, but the bipolar system had a clear advantage in blood loss, in hyponatremia events, and in catheter stay. The authors stressed the inherent potential of the new technique to become the new gold standard of the minimal invasive prostatectomy [22].

Similar to the PK system, several authors focused on the advantage of the Olympus TURis over the monopolar system in terms of complication rates. In prostates >50 mL, the hemoglobin level drop was minimal, the immediate postoperative complications were fewer, and the hospitalization and catheter stay were shorter [23]. Others underscore the limited postoperative drop in sodium level minimizing the risk of TUR syndrome [24]. All the aforementioned papers have a short follow-up period; therefore, the issue of late complications and durable results over time remains to be answered.

In a study of 136 patients with a follow-up of 3 years, the authors compared the TURis with the M-TURP [25]. In the subgroup of patients with small adenomas, both techniques yield similar results regarding the postoperative complications but in prostates >70 mL the urethral stricture rate was as high as 20% in the TURis arm and only 2.2% in the monopolar one (*P* = 0.012). Likewise, Rassweiler et al. reported on high stricture formation rates among patients treated either with the PKS or the TURis device [18]. These alarming results were not confirmed in the meta-analysis published by Omar and colleagues, in which the percentage of urethral strictures was not higher than 3.3% [13].

In our series, 8,6% of the patients developed urethral stenosis. The prolonged surgical time and perhaps the large caliber of the resectoscope sheath (26 F) might constitute the explanation for this complication. It could be assumed that, for some urethras, the resectoscope sheath may be large enough as to cause ischemia and urethral trauma. In addition, the power settings of 310 W and 170 W that we used for resection and coagulation may have produced a thermal damage to the sensitive periurethral tissue and, thus, stricture formation [14, 26]. By adjusting the working settings to a lower power level, we hope that we will be able to reduce the frequency of this complication.

The UTI rate was as high as 14.6% among large prostates and approximately 6,6% in smaller adenomas. It should be stated that we registered not only patients with febrile urogenital infection but those with asymptomatic or minimal bothersome positive urinary culture as well. Except for patients with an indwelling urethral catheter, we performed a preoperative urinary culture and we proceeded to the operation only when the results of the urine culture were negative for bacteria, or sterile. We routinely administered a cephalosporin II or an ampicillin/sulbactam regimen intravenously, 30′–60′ before and 5-6 hours after the operation. In some cases with a history of catheterization, or an estimated high risk for infection, particularly in large adenomas, we continued the treatment for 5–7 days orally. Apart from the hypothesis that large prostates may host a plethora of bacterial populations or more aggressive strains that are released into the circulation during prostatectomy and the longer operation time, no clear explanation for the high infection rate could be given.

In our opinion, the disadvantages of the TURis, including the cost of surgical loop and plasma button electrode, are counterbalanced by the short time of postoperative fluid irrigation, catheter stay, and hospitalization. Considering that the majority of patients were discharged after less than 36 hours of hospital stay, it is safe to assume that the benefit for the health care system is major. Although we have not performed an official technoeconomic study, one day less of hospital stay is translated into approximately >400€ of cost savings. The brief postoperative recovery time and the early return to work also have a profound positive effect on the individual's psychological and economic status.

A main drawback of our study is the limited number of patients, and the lack of a control group for a direct comparison of the bipolar technique against the monopolar one. Due to the short follow-up period, the long term effectiveness and the late complications of TURis are impossible to be defined in this series.

## 5. Conclusions

Treatment of BPH with the bipolar resectoscope is an effective surgical technique and seems to offer patients with large prostates surgical results equivalent to those encountered in smaller prostate volumes. Concerning the safety profile, in our series, large prostates treated with TURis are not at a higher risk for urethral stricture, but their odds to develop urogenital infections are higher compared with the smaller adenomas counterparts. Generally speaking, the percentage of postoperative strictures and infections could be considered suboptimal and should be subjected to investigation in future prospective trials. Candidates for TURis prostatectomy, irrespective of their prostatic volume, should be properly informed about the aforementioned complications before giving their consent for surgery.

## Figures and Tables

**Table 1:** Patients' characteristics at presentation.

Factor	Mean value	Range
Age (years)	71.3	46–92
Prostate volume (mL)	60.98	43–185
IPSS	18.2	7–32
QoL	3.37	2–6
*Q*_max⁡_ (mL/sec)	8.44	2–14
Residual urine (mL)	167.71	20–700
Urinary retention	6	

**Table 2:** Presentation of the operation time, catheterization time, hospital stay, and surgical results for the whole group of patients.

Factor	Mean value (range)	Percentage of change	*P* value
Operation time (minutes)	63.26 (36–151)		
Catheterization time (hours)	28.06 (16–98)		
Hospital stay (hours)	32.01 (22–75)		
IPSS^‡^ pre/post	18.2/9.57	−47.41%	0.001
QoL^‡^ pre/post	3.37/1.46	−56.67%	0.001
*Q*_max⁡_^†^ pre/post	8.44/16.97	101.07%	0.001
Residual urine^‡^ pre/post	167.71/57.08	−65.97%	0.001

^‡^Abnormal distribution: the comparison was made with the Wilcoxon matched pairs signed ranks test.

^†^Normal distribution: the comparison was made with the *t*-test (matched pairs).

**Table 3:** Overview of surgical complications for both groups of patients.

Complication	Number of patients (*n* = 93)	Percentage (%)
Urethral stricture	8	8.6%
Urinary retention	2	2.1%
Blood transfusion	1	1.0%
Urogenital infection	10	10.7%
Prolonged LUTS	9	9.6%
Prolonged hematuria	2	2.1%
Cardiovascular events	2	2.1%

**Table 4:** Comparison of the preoperative characteristics of the two groups of patients.

Variant	Group Α	Group Β	*P* value
	(*n* = 48)	(*n* = 45)	
Age^†^ (yrs)	72.3 (7.18)	71.0 (10.56)	0.631
*V*_prost_^‡^ (cm^3^)	92.1 [75–185]	51.2 [43–66]	<0.001
IPSS^†^	19.6 (6.26)	17.7 (5.39)	0.221
Qol^†^	3.7 (0.98)	3.25 (0.93)	0.093
*Q*_max⁡_^†^ (mL/sec)	6.6 (2.87)	9.0 (3.12)	0.011
RU^‡^ (mL)	162.5 [150–240]	150 [100–200]	0.386

^‡^Abnormal distribution: the median and IQR values are shown and the comparison is based on the Wilcoxon rank-sum test.

^†^Normal distribution: the mean and SD values are shown and the comparison is based on the *t*-test.

**Table 5:** Comparison of the surgical outcome, operation time, catheterization time, and hospital stay between the two groups of patients (OK).

Variant	Group Α	Group Β	*P* value
	(*n* = 48)	(*n* = 45)	
IPSS^‡^	−52.46%	−45.41%	0.934
QoL^†^	−47.57%	−60.60%	0.603
*Q*_max⁡_^†^ (mL/sec)	157.68%	116.08%	0.384
RU^‡^ (mL)	−65.03%	−65.82%	0.655
Mean operation time (minutes)^‡^	88.76	54.23	<0.001
Mean catheterization time (hours)^‡^	29.41	27.58	0.356
Mean hospital stay (hours)^‡^	34.06	31.29	0.211

^‡^Abnormal distribution: the comparison is based on the Wilcoxon rank-sum test.

^†^Normal distribution: the comparison is based on the *t*-test.

**Table 6:** Comparison of surgical complications between the two groups.

Complication	Group Α	Group Β	*P* value
	(*n* = 48): number of patients (%)	(*n* = 45): number of patients (%)	
Urethral stricture	6 (12.5%)	2 (4.4%)	0.163
Urinary retention	1 (2.1%)	1 (2.2%)	0.974
Blood transfusion	1 (2.1%)	0 (0%)	0.328
Urogenital infection	7 (14.6%)	3 (6.6%)	0.219
Prolonged LUTS/incontinence	6 (12.5%)	3 (6.6%)	0.185
Prolonged hematuria	2 (4.2%)	0 (0%)	0.166
Cardiovascular events	2 (4.2%)	0 (0%)	0.166
